# Impact of ERT and follow-up of 17 patients from the same family with a mild form of MPS II

**DOI:** 10.1016/j.clinsp.2022.100082

**Published:** 2022-07-23

**Authors:** Bruno de Oliveira Stephan, Caio Robledo Quaio, Gustavo Marquezani Spolador, Ana Carolina de Paula, Marco Antônio Curiati, Ana Maria Martins, Gabriela Nunes Leal, Artur Tenorio, Simone Finzi, Flavia Teixeira Chimelo, Carla Gentile Matas, Rachel Sayuri Honjo, Debora Romeo Bertola, Chong Ae Kim

**Affiliations:** a Instituto da Criança, Hospital das Clínicas, Faculdade de Medicina, Universidade de São Paulo (HCFMUSP), São Paulo, SP, Brazil; b Centro de Referência em Erros Inatos do Metabolismo (CREIM), Universidade Federal de São Paulo (UNIFESP), São Paulo, SP, Brazil; c Oftalmologia, Hospital das Clínicas, Faculdade de Medicina, Universidade de São Paulo (HCFMUSP), São Paulo, SP, Brazil; d Fonoaudiologia da Faculdade de Medicina, Universidade de São Paulo (FMUSP), São Paulo, SP, Brazil

## Abstract

**Background:**

Mucopolysaccharidosis type II, also known as Hunter syndrome, is a rare X-linked recessive disorder caused by deficiency of the lysosomal enzyme Iduronate-2- Sulfatase (IDS), leading to progressive accumulation of Glycosaminoglycans (GAGs) in several organs. Over the years, Enzyme Replacement Therapy (ERT) has provided significant benefits for patients, retarding the natural progression of the disease.

**Results:**

The authors evaluated 17 patients from the same family with a mild form of MPS type II; the proband had developed acute decompensated heart failure refractory to clinical measurements at 23 years and needed a rather urgent heart transplant; however, he died from surgical complications shortly after the procedure. Nevertheless, subsequent to his tragic death, 16 affected male relatives were detected after biochemical tests identifying the low or absent activity of the IDS enzyme and confirmed by molecular analysis of the IDS gene. Following diagnosis, different options of treatment were chosen: 6 patients started ERT with Elaprase® (Idursulfase) soon after, while the other 10 remained without ERT. Eventually, 4 patients in the latter group began ERT with Hunterase® (Idursulfase Beta). None presented adverse effects to either form of the enzyme. Among the 6 individuals without any ERT, two died of natural causes, after reaching 70 years. Despite the variable phenotype within the same family (mainly heart dysfunctions and carpal tunnel syndrome), all 14 remaining patients were alive with an independent lifestyle.

**Conclusion:**

Here, the authors report the variable progress of the disease with and without ERT in a large Brazilian family with a slowly progressive form of MPS II, harboring the same missense variant in the IDS gene.

## Highlights

• Clinical variability within a large family with a slowly progressive form of MPS II.
• Despite being safe, ERT with α or β-Idursulfase might not prevent disease progression.

## Introduction

Mucopolysaccharidosis type II (MPS II, OMIM 607014; also known as Hunter syndrome) is a rare X-linked recessive disorder caused by a deficiency of the lysosomal enzyme Iduronate-2-Sulfatase (IDS), leading to the progressive accumulation of Glycosaminoglycans (GAGs) in several organs. The chronic deposition of undegraded GAGs in the connective tissue contributes to the progressive clinical course of the syndrome with a variable degree of severity and life expectancy. Increased urinary excretion of dermatan and heparan sulfate, low or absent activity of the IDS enzyme, and molecular analysis of the IDS gene may confirm the diagnosis.^1^, ^2^, ^3^, ^4^

The authors have previously reported a large Brazilian family with 17 individuals affected by a very slowly progressive form of MPS II, all harboring the p. A77D mutation in the IDS gene.[5] Diagnosis of this family was only possible because the proband had developed acute decompensated heart failure refractory to clinical measurements and required a heart transplant. His clinical evaluation before the procedure showed mild coarse face, hepatomegaly, and joint stiffness of the shoulders; biochemical assays showed an increased excretion of GAGs in the urine and a deficiency of IDS activity in leukocytes, confirming the diagnosis of MPS II. The first heart transplant in a patient with MPS II was then performed using a bicaval orthotopic heart transplantation technique, but unfortunately, the patient died from primary left ventricular failure 2 days after the surgery.[6] In the proband's family, 16 other affected individuals with the mild form of MPS II and 14 female carriers of the trait were discovered after this episode.

The severe form of MPS II is characterized by facial dysmorphism, short stature, hepatosplenomegaly, hernias, stiff joints and contractures, cardiac valve disease, obstructive respiratory complications, chronic diarrhea, hearing loss, communicating hydrocephalus, cognitive impairment, and death by progressive airway obstruction or cardiac dysfunction between the ages of 10 and 15 years.[1,2,7] However, the mild (or slowly progressive) form of MPS II is generally characterized by a normal life expectancy; the most serious complications in these patients are usually secondary to heart defects, which may lead to premature death in some cases.

The objective of this article is to describe the follow-up of this large family, divided into members with two different enzyme replacement therapies and others without Enzyme Therapy (ERT).

## Method

### Clinical information and biochemical analysis

This study was conducted on 17 related patients from a single-family in which the diagnosis of MPS II was established after the demonstration of concomitant increased urinary excretion of dermatan and heparan sulfate, the low or absent activity of the IDS enzyme, and molecular analysis of the IDS gene.

All patients were followed by a clinical geneticist. Each patient received genetic counseling and signed a written informed consent form. If the patient was underage, the parents or legal representative provided written consent. This study was approved by the ethics committee of our institution.

Prospective data have been collected from these patients over the last decade.

### Molecular analysis

Genomic DNA was isolated from peripheral blood lymphocytes; exonic regions and exon-intron boundaries of the IDS gene were then amplified by PCR and sequenced using the Sanger technique and standard protocols.

### Variant classification

Variants were interpreted and classified according to the ACMG variant interpretation guideline.[8] The case was only considered as diagnosed when pathogenic or likely pathogenic variants were observed in a gene that was associated with the phenotype in the studied individual, with compatible zygosity, and in an adequate inheritance pattern.

The 1000 Genomes project database (http://www.1000genomes.org), including all human genetic variations from the dbSNP short genetic variations database (https://www.ncbi.nlm.nih.gov/snp/), and the ExAC Browser of the Exome Aggregation Consortium (now part of the Genome Aggregation Database), which provides a dataset spanning over 60,000 unrelated individuals (http://exac.broadinstitute.org or https://gnomad.broadinstitute.org/), were both used to evaluate the polymorphic status of the identified genetic alterations.

### Enzyme replacement therapy and follow-up protocol

Following diagnosis in 16 males from the family of the proband, 10 of them started ERT: 6 received Idursulfase (Elaprase®) and the other 4, Idursulfase Beta (Hunterase®). The other 6 patients did not receive any ERT; two died of natural causes, after reaching 70 years.

## Results

### Clinical and biochemical data

The authors report the available clinical and biochemical information collected for each patient on the tables attached (Tables 1 and 2). The previously asymptomatic proband had an ordinary life until 23 years of age, making it possible for him to graduate from university. He was then admitted to cardiologic intensive care unit due to acute decompensated heart failure, which advanced drastically within 1 month. A physical exam at that time showed normal height, weight, and occipital frontal circumference (OFC: 56 cm), but also revealed classical features of the disease previously unnoticed, such as the mild coarse face, hepatomegaly, and joint stiffness of the shoulders. Biochemical assays then showed an increased excretion of GAGs in the urine as well as a deficiency of IDS activity in leukocytes, confirming the diagnosis of MPS type II. At this point, an echocardiogram demonstrated thickened valves with severe mitral regurgitation, severe left ventricular dilatation, and severe ventricular dysfunction. Since his heart failure was refractory to clinical management, a heart transplant was indicated. Unfortunately, the patient died of primary graft failure 2 days after the surgery. Postmortem histopathological analysis of the myocardium by electron microscopy revealed that GAGs accumulated in the interstitium of the myocytes.[6]

**Fig. 1:**
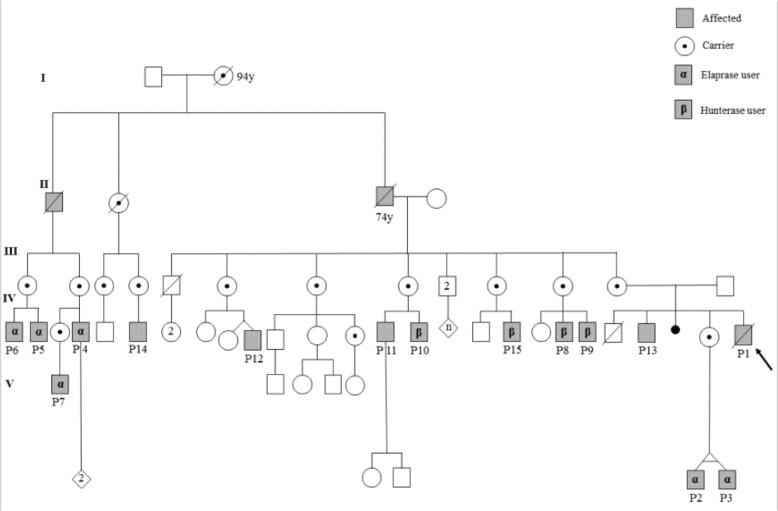
(Pedigree).

Following the proband's diagnosis and almost immediate death, 16 other affected individuals and 14 female carriers were identified in a family pedigree study (Fig. 1). Even though most of the male relatives were also allegedly asymptomatic, clinical, biochemical, and molecular tests were conducted among the living family members. At the time, the authors examined 14 affected patients, including 9 adults over 18 years of age, 3 teenagers, and a one-year-old pair of identical twins. The age of diagnosis was between 8 months and 35 years, with a mean of 18.6 years.

Anthropometric measurements, including Body-Mass Index (BMI), were within normal percentiles for most of the patients, although two adults presented short stature (heights of 153 and 151 cm from P5 and P15, respectively) and another one showed signs of microcephaly (P4 had an OFC > 60 cm). Mild coarse facies proved to be a universal finding, whereas hepatomegaly was clinically observed only in the proband (probably secondary to acute decompensated heart failure), although 60% presented hepatosplenomegaly at some point of life. No one presented lumbar gibbus, claw hands, or deformities other than mild articular restriction, such as stiffness of shoulders and carpal tunnel syndrome.

Despite only the proband becoming frankly symptomatic and evolving to cardiac failure, all except one (P6) of the living 14 men presented some degree of heart damage, mainly valvulopathies: the tricuspid valve was involved in six patients; the aortic valve in nine; and the mitral valve in twelve.

Among surgical interventions, resection of adenoid and/or tonsil tissues was by far the most common procedure. Since airway obstruction is progressive and frequent in MPS II,[9] surgical correction of hypertrophy of the adenoid and/or tonsil tissues was also necessary for 9 patients (7 of them during childhood). Likewise, hernias (either umbilical or inguinal) are very common in this disease, demanding the surgical intervention at some point in the life of 10 individuals.

Articular restriction was noteworthy as well, affecting the shoulders in most individuals. In the mild form of MPS II, joint contractures usually involve the upper extremities more prominently than the lower extremities.

As expected for MPS II patients, none of the individuals had corneal opacities. However, retinopathies were reported: two patients (P10 and P12) presented retinitis pigmentosa and underwent peripheral iridotomy due to Primary Angle Closure (PAC), while one other (P13) showed optic nerve swelling, which responded well to clinical treatment. In addition, hypermetropia was present in 6 patients, with retinal detachment secondary to trauma occurring in one of them (P8).

Neurologic signs of the disease were suspected in only one patient (P11), who presented a remarkable speech delay (as he learned to talk only at 5 years); however, his condition was later attributed to the repeated crisis of otitis media and a severe hearing disability. Of the 14 patients, 8 underwent assessment of the peripheral and central auditory pathways; 62.5% had mild to moderate hearing loss, with the sensorineural type being the most frequent finding. Regarding the assessment of the central auditory pathway, 87.5% showed changes.

All adults had normal cognitive development (including P11), making it possible for them to graduate from normal school and perform satisfactorily in their ordinary jobs. As expected, the 14 female carriers did not present any signs of disease activity.

Initial urinary levels of GAGs varied from 108 to 683 μg/mg creatinine (normal: 13–45), with a mean of 239.8 μg/mg; for those patients who received ERT, levels reached a mean of 95.1 μg/mg after its introduction. Enzymatic dosage through IDS activity in leukocytes ranged from undetectable to 4.3 nmoL/4h mg protein, which represents a maximum of 13.8% of the minimal value usually seen in the normal population (value of reference: 31–110 nmoL/4h mg protein).

### IDS molecular analysis

Segregation analysis of this family showed a perfect correlation, which was highly suggestive of cosegregation of this variant in affected individuals. Female patients suspected of being obligatory carriers, as well as patients presenting biochemical evidence of diminished IDS enzyme activity, were submitted to molecular analysis, all of them harboring the same hemizygous variant (p.A77D) in the IDS gene.

### Variant classification

First, the variant was located in a mutational hotspot (PM1); additionally, this was a missense in a gene with a low rate of benign missense variants, for which such a finding is a well-known mechanism of disease (PP2). It was also in perfect cosegregation with the disease in multiple affected family members (PP1), and the phenotype was highly specific for genes as well (PP4). Finally, not only was the variant not found in control databases (PM2), but also multiple lines of computational evidence supported a deleterious effect (PP3). Hence, according to the ACMG variant interpretation guideline,[8] the variant was classified as likely pathogenic.

### Enzyme replacement therapy and follow-up protocol

Among the 10 patients who received ERT (Table 1), 6 were given Idursulfase (Elaprase®) and, the other 4, were given Idursulfase Beta (Hunterase®). The age of introduction of treatment was 1 to 26 and 27 to 37 years, respectively. In accordance with most of the reviews,[4] there were no significant differences between the medications chosen. Out of the 6 patients without ERT, two elderly died of natural causes.

**Table 1:** Patients with ERT (Elaprase × Hunterase).

Patient	Clinical findings	Type of enzyme/ duration of ERT	ERT starting age	GAGs (μg/mg creatinine) before/ after ERT	Echocardiogram before/ after ERT	Visceral before/ after ERT	Skeletal before/ After ERT
**P2**	Sleep apnea; Recurrent respiratory tract infections, umbilical hernia	Elaprase®/ 12 years	1 year and 5 months old	683 (2009)/ 165 (2012)	Normal/ =	Hepato and splenomegaly/ =	Disostosis multiplex/ NA
**P3**	Sleep apnea; adenoid and tonsil hypertrophy, umbilical hernia; articulary restriction	Elaprase®/ 12 years	1 year and 5 months old	648 (2009)/ 129 (2012)	Normal/ Mild thickening of mitral valve without dysfunction	Hepato and splenomegaly/ =	Disostosis multiplex/ NA
**P4**	Adenoid and tonsil hypertrophy; umbilical hernia; limb paresthesia and pain	Elaprase®/ 11 years	26-years-old	NA/ 82 (2015)	Mitral and tricuspid insufficiency; left ventricular hypertrophy / NA	Hepatomegaly/ =	NA/ Femoral cyst measuring 3.1 cm
**P5**	Rhinitis, tonsil hypertrophy, recurrent ear infection	Elaprase®/ 12 years	7-years-old	NA	NA/ Aortic and mitral mild insufficiency	Hepatomegaly/ =	NA
**P6**	Recurrent respiratory infection, lactose intolerance, chronic diarrhea	Elaprase®/ 12 years	3-years-old	NA	NA/ Normal	Hepatomegaly/ =	NA
**P7**	Articulary restriction	Elaprase®/ 12 years	8-years-old	219 (2008)/ 102 (2015)	Moderate aortic insufficiency/ Aortic valvar dysplasia and regurgitation; left ventricular dilatation	Splenomegaly/ =	Normal/ NA
**P8**	Recurrent otitis media, carpal tunnel syndrome, articulary restriction, obstructive lung disease	Hunterase®/ 5 years	29-years-old	120 (2010) / 54 (2017)	Mild mitral and tricuspid insufficiency/ =	NA / Alteration of liver texture	Disostosis multiplex; Fusion of C2-C3 posterior arcs/ =
**P9**	Sensorineural hearing loss, hyperopia, carpal tunnel syndrome articulary restriction	Hunterase®/ 4 years	29-years-old	157 (2009)/ 30 (2019)	Aortic Insufficiency mild to moderate/ Mild left ventricular hypertrophy; Diastolic disfunction of right and left ventricle; Thickening of mitral and aortic valves; Mild aortic and mitral insufficiency	NA/ Normal	NA/ Disostosis multiplex
**P10**	Tonsil hypertrophy, inguinal hernia, carpal tunnel syndrome, recurrent otitis media, sensorineural hearing loss	Hunterase®/ 5 years	37-years-old	170 (2015)/ 137 (2018)	NA/ Normal with mild tricuspid valve reflux	NA	Disostosis multiplex/ =
**P15**	Chiari I, sleep apnea. hyperopia, tonsil hypertrophy, umbilical hernia, carpal tunnel syndrome, articulary restriction	Hunterase®/ 5 years	26-years-old	151 (2009)/ 62 (2017)	Mild thickened and reflux of mitral and aortic valve, mild tricuspid reflux/ Mild sistolic miocardic deformation of left ventricle	Solid nodule in the right hepatic lobe/ Compatible with hemangioma	Disostosis multiple/ =

NA, Not available; =, Same result.

Even though the echocardiographic and ophthalmologic findings seemed to be more severe in the second group (Hunterase®), it is important to point out that the patients in the first group (Elaprase®) were not only considerably younger but also received medication for far more time (mean total time of treatment of 11.8 versus against 4.75 years, respectively).

In fact, these older patients opted to initiate ERT primarily due to worsening nocturne vision; at the time, due to bureaucratic reasons, Idursulfase Beta (Hunterase®) was available and easier to access than Idursulfase (Elaprase®). Nevertheless, in accordance with most of the reviews,[4] none of the men under ERT presented any relevant adverse effect to either of the two enzymes, to date (after more than a decade of treatment for most).

Finally, the authors detected some minor alterations (mild mitral insufficiency) in the early stages of ERT (at the age of 4) of our pair of identical twins (P2 and P3) in the first group. However, after their last appointment (at the age of 11), valvopathy persisted in only one of the twins (P3), while the other (P2) revealed a rather normal echocardiogram. Nevertheless, despite this peculiarity, both of them (who are now 12 years old) always had an unremarkable clinical evaluations.

## Discussion

This large Brazilian family with 17 individuals displayed a very slowly progressive form of MPS II, with normal cognitive development and preserved quality of life. The diagnosis of this condition in otherwise asymptomatic people was only possible because of the proband's acute decompensated heart failure. This disease has been traditionally classified into slowly progressive and severe subtypes, although variations in severity between these two extremes have been observed.

Cardiovascular involvement was the most common serious complication of the disease in this family, although the proband was the only person to present with major cardiovascular symptoms. The fast progression of his heart failure at the young age of 23 years was conspicuous. The failure of the clinical management plus the severity of heart involvement determined his need for a heart transplant, which was performed for the first time in the context of MPS II. Unfortunately, the surgical approach failed in its objective, as the patient died of primary graft failure on the second postoperative day.

A remarkable variability of heart involvement was observed among affected individuals. The proband's 28-year-old brother had only mild aortic regurgitation, while his 19-year-old cousin had a normal echocardiogram. Interestingly, although other family members had severe valvar lesions in echocardiography studies, they had no complaints or significant symptoms of heart failure. In the literature, symptomatic individuals constitute more than 80% of all MPS II patients, whereas valvar disease may be found in up to 60%.[16] Considering the importance of cardiovascular involvement in MPS II, physicians must always be aware of periodically monitoring heart complications during the follow-up of these patients through comprehensive clinical examination and routine echocardiography, always keeping in mind that some degree of the disease is usually expected, even in healthy patients.

Although life expectancy is close to normal in less severely affected individuals, as previously reported,^10^, ^11^, ^12^ authors believe that sensorineural hearing impairment is progressive and relates to cochlear fragility due to cell cilia dysfunction.^13^, ^14^, ^15^ Those findings emphasize the importance of referring these patients for an audiological evaluation, as well as monitoring from an auditory perspective. In fact, the two affected seniors the authors identified died of natural causes at advanced ages (both older than 70 years old).

According to most sources, there seems to be no clear genotype-phenotype correlation in MPS II. Of the 650 variants in IDS reported, nearly half are missense mutations,[4] similar to the one reported here. However, it is important to point out that the severe forms of the disease are usually associated with mutations that result in the complete absence of enzyme activity, such as nonsense and splicing variants, as well as large CNVs or complex rearrangements.

To date, the specific mutation of this family (p.A77D) remains unreported by other studies or laboratories, according to databases such as the LOVD (Leiden Open Variant Database). In addition, analysis from over a dozen silico prediction platforms (including REVEL and MutationTaster) indicates that this variant indeed has a disease-causing effect.

Regarding treatment, for many decades following Hunter's initial description of MPS II (back in 1917), there was no effective therapy for the disease, and management was restricted to a palliative approach. Hematopoietic stem-cell transplantation, which has been a major advance in the treatment of other forms of MPS in recent decades, seems to have a poor outcome for MPS II patients. Various forms of gene therapy (from viral vectors to substrate reduction therapies), although promising, remain distant from the clinical scenario for MPS II [4] thus far.

Fortunately, in the last fourteen years, the advent of Enzyme Replacement Therapy (ERT) has changed the clinical approach to MPS II, adding the possibility of improving the degradation and excretion of GAGs. Given that it postpones the systemic development and natural history of the disease, ERT has become the standard of care for MPS II and has reinforced the importance of early diagnosis. However, many doubts about its application remains unresolved.

Considering that urinary levels of GAGs do not seem to have a clear direct correlation with the clinical severity of disease and that ERT for MPS II (or other types of mucopolysaccharidosis) frequently does not normalize GAG levels,[17] therapeutical response tends to be quite difficult to measure through laboratory parameters. Additionally, long-term benefits are somewhat limited in patients with the severe phenotype, because it is increasingly evident that plasma infusions will not penetrate the Blood-Brain Barrier (BBB) and thus are unable to prevent the advance of the disease in the Central Nervous System (CNS).[18,19] Finally, the age at which ERT should be initiated and whether asymptomatic individuals presenting the mild form of MPS II must be treated remain uncertain.[4,20,21]

In this scenario, it is important to acknowledge that the two types of ERT available for MPS II, Idursulfase (Elaprase®) and Idursulfase Beta (Hunterase®), were not distributed randomly to the present study's patients; as explained above, some of the individuals opted to initiate ERT only at adult age, when, due to nonmedical reasons, Idursulfase Beta (Hunterase®) became easier and faster to access.

Nevertheless, other than a few differences that cannot be discarded from the natural progression of the disease, the authors could not find other significant differences between the two sets of patients with different types of enzymes. Indeed, as most data suggest 21, 22, 23 both drugs appear overall equally efficient and well-tolerated.

Finally, the authors admit that the progression of the disease in the four patients of this family without ERT was rather uneventful (Table 2), despite their advanced age (all over 40 years). However, in this scenario, one must point out that the follow-up of such individuals was neglected due to a lack of more careful support because this group received health services less frequently. Nevertheless, despite the variable phenotype (mainly heart dysfunctions and carpal tunnel syndrome), all 14 remaining living patients remain clinically healthy and have an independent lifestyle.

**Table 2:** Patients without ERT.

Patient	Clinical findings	GAGs (μg/mg creatinine)	Echocardiogram	Visceral	Skeletal
**P1**	Cardiac failure, umbilical, and inguinal hernia, articulary restriction.	NA	Dilated Cardiomyopathy with valvar thickening; aortic and mitral insufficiency (23 years)	Hepatomegaly	NA
	†Deceased at 23 years old after cardiac transplantation.				
**P11**	Recurrent otitis media, sensorineural hearing loss, hyperopia, adenoid and tonsil hypertrophy, inguinal hernia, articulary restriction	113 (last dosed in 2017)	Thickened mitral and aortic valves with mild insufficiency; mild insufficiency of tricuspid valve (33 years)	Splenomegaly	Disostosis multiplex
**P12**	Sleep apnea, dyspnea, anxiety, hyperopia, umbilical and inguinal hernias, carpal tunnel syndrome, articulary restriction	159 (last dosed in 2017)	Important mitral insufficiency, moderate aortic insufficiency, and mild tricuspid insufficiency (36 years)	NA	Disostosis multiplex
**P13**	Sensorineural hearing loss, hyperopia, papilledema, tonsil hypertrophy, umbilical and inguinal hérnia, carpal tunnel syndrome	126 (last dosed in 2016)	Mitral and aortic valve insufficiency; concentric left ventricular hypertrophy (34 years)	Hepato and splenomegaly	Disostosis multiplex
**P14**	Tonsil hypertrophy, umbilical and inguinal hérnia, articulary restriction	114 (last dosed in 2010)	Mitral valve insufficiency; left ventricular hypertrophy (32 years)	Hepatomegaly (child)	NA

NA, Not available; =, Same result.

## Conclusion

Considering the variability in the progression and the clinical phenotype of MPS II, the authors observed that even individuals of the same family, presenting the same identical mutation, manifested the slowly progressive form of the disease quite differently. As mentioned above, the p.A77D missense mutation of the IDS gene described here has not been reported and probably allowed residual enzyme activity to prevent the severe form of MPS II, sparing most patients of neurological impairment or any other major impairment.

After a careful analysis of the present familial cases with 17 individuals displaying a very slowly progressive form of the disease, the practical impact of ERT remains uncertain. For the 10 individuals receiving ERT, both types of Idursulfase showed similar results and no adverse effects, while in the other 4 living patients without any ERT, the progression of the disease continued rather slowly. Overall, the difference in the clinical outcome between those groups was minimal.

Nevertheless, considering the tragic outcome of the proband, it is the present authors’ opinion that such a form of therapy should always be offered. Therefore, as most reviews suggest, such decisions should still be secondary to the clinical condition of each patient.

In conclusion, in this large family with a very slowly progressive form of MPS II and a variable degree of clinical manifestations, it is noteworthy that several affected individuals have remained asymptomatic even at advanced ages without ERT.

All authors have reviewed and approved the final version of the manuscript.

## Authors’ contributions

Bruno de Oliveira Stephan: Contributed to the conception and design of the article, as well as the analysis and interpretation of data.

Gustavo Marquezani Spolador: Contributed to the conception and design of the article, as well as the analysis and interpretation of data.

Caio Robledo Quaio: Contributed to the conception and design of the article, as well as the analysis and interpretation of data.

Ana Carolina de Paula: Contributed to the analysis and interpretation of data, especially concerning the clinical and genetic evaluation of the patients.

Marco Curiati: Contributed to the analysis and interpretation of data, specially concerning the clinical and genetic evaluation of the patients.

Ana Maria Martins: Contributed to the analysis and interpretation of data, specially concerning the clinical and genetic evaluation of the patients.

Gabriela Nunes Leal: Contributed to the analysis and interpretation of data, specially concerning the echocardiographic evaluation of the patients.

Artur Tenorio: Contributed to the analysis and interpretation of data, specially concerning the ophthalmological evaluation of the patients.

Simone Finzi: Contributed to the analysis and interpretation of data, specially concerning the ophthalmological evaluation of the patients.

Flavia Teixeira Chimelo: Contributed to the analysis and interpretation of data, specially concerning the audiological evaluation of the patients.

Carla Gentile Matas: Contributed to the analysis and interpretation of data, specially concerning the audiological evaluation of the patients.

Rachel Sayuri Honjo: Contributed to the draft and critical revision of the article.

Debora Romeo Bertola: Contributed to the draft and critical revision of the article.

Chong Ae Kim: Contributed to the draft and critical revision of the article.

## Ethics approval and consent to participate

All patients were followed by a clinical geneticist. A written consent form was obtained from all participants. This study was approved by the ethics committee of our institution. There are no conflicts of interest to disclose.

## Consent for publication

Each patient received genetics counseling and signed a written consent form. If the patient was underage, the parents or legal representative provided a written consent form.

## Availability of data and materials

Prospective data have been collected from these patients over the last decade. Please contact the author for data requests.

## Funding

Funding information is not applicable.

## Conflicts of interest

The authors declare no conflicts of interest.
